# A Handheld Ultrasound Device Can Predict Constipation with Rectal Fecal Retention in a Palliative Care Setting

**DOI:** 10.3390/diagnostics14151626

**Published:** 2024-07-28

**Authors:** Atsushi Yamamoto, Takaomi Kessoku, Tomoki Ogata, Tsumugi Jono, Kota Takahashi, Kosuke Tanaka, Ko Suzuki, Yuma Takeda, Anna Ozaki, Yuki Kasai, Naoki Okubo, Michihiro Iwaki, Takashi Kobayashi, Noboru Misawa, Tsutomu Yoshihara, Akihiro Suzuki, Akiko Fuyuki, Sho Hasegawa, Kento Imajo, Noritoshi Kobayashi, Masaru Matsumoto, Nao Tamai, Hiromi Sanada, Shunsuke Oyamada, Yasushi Ichikawa, Atsushi Nakajima

**Affiliations:** 1 Department of Gastroenterology and Hepatology, Yokohama City University Graduate School of Medicine, Yokohama 236-0004, Japan; atsushi.y.0410@gmail.com (A.Y.); tomo1993baske@gmail.com (T.O.); tsumuchon@gmail.com (T.J.); takahashi1700pk9@gmail.com (K.T.); tanaka.kos.la@yokohama-cu.ac.jp (K.T.); suzuki.ko.tg@yokohama-cu.ac.jp (K.S.); anx0513ro@hotmail.com (A.O.); kasai.yuk.wd@yokohama-cu.ac.jp (Y.K.); t206009g@yokohama-cu.ac.jp (N.O.); michihir@yokohama-cu.ac.jp (M.I.); tkbys@yokohama-cu.ac.jp (T.K.); nobomisa@yokohama-cu.ac.jp (N.M.); t_yoshi@yokohama-cu.ac.jp (T.Y.); aksuzuki@yokohama-cu.ac.jp (A.S.); fuyukia@yokohama-cu.ac.jp (A.F.); t166064d@yokohama-cu.ac.jp (S.H.); kento318@yokohama-cu.ac.jp (K.I.); nakajima-tky@umin.ac.jp (A.N.); 2 Department of Gastroenterology, Fujisawa Syonandai Hospital, Fujisawa 252-0802, Japan; 3 Department of Palliative Medicine, International University Health and Welfare, Narita Hospital, Narita 286-8520, Japan; 4 Department of Gastroenterology, International University Health and Welfare, Narita Hospital, Narita 286-8520, Japan; 5 Department of Gastroenterology, Yokosuka General Hospital Uwamachi, Yokosuka 238-0017, Japan; 6 Department of Palliative Medicine, Yokohama City University, Yokohama 236-0004, Japan; takeda14@yokohama-cu.ac.jp (Y.T.); yasu0514@yokohama-cu.ac.jp (Y.I.); 7 Department of Oncology, Yokohama City University, Yokohama 236-0004, Japan; norikoba@yokohama-cu.ac.jp; 8 Department of Palliative Care, Shin-Yurigaoka General Hospital, Kawasaki 215-0026, Japan; 9 Department of Gastroenterology, Shin-Yurigaoka General Hospital, Kawasaki 215-0026, Japan; 10 School of Nursing, Ishikawa Prefectural Nursing University, Kahoku 929-1210, Japan; matumoto@ishikawa-nu.ac.jp (M.M.); sanadah@ishikawa-nu.ac.jp (H.S.); 11 Department of Nursing, Yokohama City University Graduate School of Medicine, Yokohama 236-0004, Japan; tamai.nao.tx@yokohama-cu.ac.jp; 12 Department of Biostatistics, JORTC Data Center, Tokyo 116-0013, Japan; shunsuke.oyamada@jortc.jp

**Keywords:** constipation, fecal retention, palliative care, ultrasonography

## Abstract

Although handheld ultrasound devices (HUDs) are commonplace, their ability to diagnose fecal retention (FR) remains unclear. This prospective observational study examined HUDs’ usefulness in diagnosing FR in patients with constipation in a palliative care setting. Between 10 December 2020 and 30 June 2022, we compared rectal ultrasonographic findings obtained using HUDs with clinical manifestations in 64 males and 70 females (48%, 52%, 68 ± 11 years old) with constipation who had undergone computed tomography (CT). FR was diagnosed using a HUD and compared with CT and digital rectal examination (DRE) results. In total, 42 (31%), 42 (31%), and 41 (31%) patients were diagnosed using HUDs, CT, and DRE, respectively. Thirty-nine (93%) patients in the CT group were also diagnosed with FR using HUDs. A total of 89 of 92 patients with a negative CT diagnosis also had a negative HUD diagnosis. Among the 41 patients in the DRE group, 37 were also diagnosed with FR using HUDs. Among 93 patients with a negative DRE diagnosis, 86 had a negative HUD diagnosis. The sensitivity, specificity, positive predictive value, and negative predictive value of HUDs for CT were 93%, 97%, 93%, and 97%, respectively. Those of HUDs for DRE were 88%, 94%, 86%, and 95%, respectively. The concordance rates for FR diagnosis were 128/134 for CT and HUDs and 123/134 for DRE and HUDs. HUD was useful for diagnosing FR in this setting. HUDs could provide valuable support for appropriate treatment selection. Developing a constipation treatment algorithm based on rectal ultrasonographic findings is warranted in the future.

## 1. Introduction

Chronic constipation is common among older adults, with a prevalence of 16% in adults and 33.5% in those aged 60 years and older [1]. However, in nursing home residents, this prevalence is as high as 50% [2]. Additionally, individuals of low socioeconomic status are more likely to become constipated than the general population [3,4]. In recent years, it has been suggested that chronic constipation is associated with an increased risk of mortality and cardiovascular events [5]; for example, a cohort study of Japanese subjects reported that decreased defecation frequency was associated with the risk of cardiovascular disease [6]. This highlights the need for improved knowledge and diagnostic techniques for treating chronic constipation.

As a frequent complication in patients with cancer receiving palliative care (rates of 30–90%), constipation interferes with pain treatment and reduces the patient’s quality of life [7,8]. Palliative care is provided to patients facing serious life-limiting illnesses. Many of these patients have a poor overall performance status and limited communication abilities; however, the diagnosis of constipation is primarily based on an interview using the Rome IV criteria, which can be problematic for the patients [9]. In addition, to diagnose constipation and fecal retention (FR), a physical examination and imaging—namely, digital rectal examination (DRE) abdominal radiography and computed tomography (CT)—are used, which often require the patients to be positioned in uncomfortable positions and environments [10,11,12].

In the past, oral and transanal treatments were performed without evidence if feces were not produced; this was done irrespective of whether the patient complained of symptoms. In particular, enemas are ineffective if there is no FR; however, the treatment was performed without checking for FR, resulting in unnecessary medical costs owing to unnecessary enemas and risking serious complications such as intestinal perforation and hemolysis. Therefore, visualization of FR is important to reduce unnecessary fecal procedures and to establish evidence-based transanal treatment.

Ultrasonography (US) is inexpensive, noninvasive, and does not involve radiation [13,14]. Specifically, abdominal US helps to diagnose constipation and evaluate treatment efficacy within a clinical setting [15,16]. In recent years, the use of point-of-care ultrasound (POCUS) using a handheld ultrasound device (HUD) by physicians or nurses not specialized in US has rapidly increased [17]. Such handheld devices allow medical professionals to monitor patients at the bedside and make prompt decisions about the appropriate care measures. Any member of a multidisciplinary team can perform POCUS to observe FR and evaluate constipation. However, HUDs’ usefulness in diagnosing FR in patients with constipation in palliative care settings remains unclear. Therefore, we investigated the usefulness of HUDs for correctly diagnosing FR in patients with constipation within the palliative care setting.

## 2. Materials and Methods

Patients with and without cancer who required symptom management and end-of-life care in a palliative care setting were evaluated. The following parameters were studied: (1) frequency of FR in patients with constipation attended to by the palliative care team (PCT), (2) diagnosis of FR using HUDs compared with the gold-standard technique, and (3) identification of the patients’ background factors associated with FR. The palliative care team consisted of physicians, nurses, pharmacists, physical therapists, clinical psychologists, and social workers.

### 2.1. Ethics

This study was conducted in accordance with the principles of the Declaration of Helsinki and approved by the ethics committee of Yokohama City University Hospital on 8 October 2019 (approval number: B190800009). Written informed consent for participation in the study was obtained from all participants prior to the study. This study was registered as UMIN000042704 (University Hospital Medical Information Network) on 9 December 2020.

### 2.2. Study Design

This prospective observational single-center study was conducted at Yokohama City University Hospital between 10 December 2020 and 30 June 2022. Inpatients referred to the PCT at a tertiary-care university hospital were included. Patient characteristics and constipation symptoms were recorded at enrollment using a questionnaire. This article was written in accordance with the STARD (Standards for Reporting Diagnostic Accuracy Studies) checklist [18].

### 2.3. Patients

Patients referred to the PCT were included if they reported constipation as a subjective symptom during symptom screening at the time of referral. A total of 134 inpatients (64 men, 70 women; mean age, 68 ± 11 years) who were newly registered with our PCT during the study period were enrolled. Inclusion criteria were patients with and without cancer who requested palliative care. Exclusion criteria were patients (1) with a surgical stoma, (2) who could not provide consent, (3) with an unknown medical history, or (4) with indwelling urinary catheters.

### 2.4. Diagnosis of Fecal Retention

Two expert gastroenterologists diagnosed FR by CT and DRE. Both CT and DRE are the gold-standard methods used for FR diagnosis. FR was diagnosed based on palpation of the fecal mass and rectal dilatation during DRE. The HUD was used within 1 h after the CT scan and DRE to minimize the likelihood of a change in stool retention status.

### 2.5. Questionnaire

Chronic constipation was defined as constipation with symptoms presenting for at least 6 months while meeting at least two of the symptoms in the following criteria for 3 months previously: straining during more than 25% of defecations, lumpy or hard stools in more than 25% of defecations, sensation of incomplete evacuation after more than 25% of defecations, difficulty in evacuation during more than 25% of defecations, manual assistance during more than 25% of defecations, and spontaneous bowel movements (SBMs) fewer than 3 times per week. Each diagnosis of functional constipation was assessed according to the Rome IV criteria [9] by an expert gastroenterologist.

Stool consistency was scored using the Bristol stool form scale (BSFS), which comprises seven different categories and evaluates the shape of the stool as follows: types 1 and 2 indicate “constipation”, types 3–5 indicate “normal defecation”, and types 6 and 7 indicate “diarrhea [19].” The sense of incomplete evacuation and the defecation desire were determined by two answers, either “no” or “yes”.

### 2.6. Position

US was performed in the supine position.

### 2.7. Imaging Using HUD

**Figure 1:**
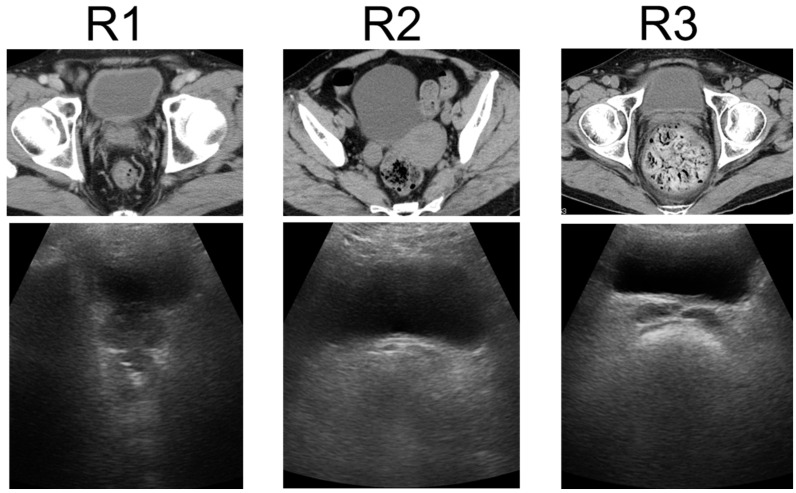
Ultrasonographic rectal findings. Transverse US images show no FR or gas in the rectum as no reflection, FR as a half-moon-shaped moderate reflection with an acoustic shadow, and hard FR as a crescent-shaped strong reflection with an acoustic shadow. US findings of the rectum were classified as R1 (no FR), R2 (FR), or R3 (hard FR). US: ultrasonography, FR: fecal retention.

US was performed using a wireless handheld ultrasound system (FWU-1 (iViz air); Fujifilm, Tokyo, Japan)) with a convex probe (2–5 MHz). This study compared the US findings of the rectum using portable ultrasound devices with the patient’s clinical manifestations. Ultrasound techniques for the rectum were finalized at a consensus meeting [20]. Transverse US images show FR in the rectum as a half-moon-shaped moderate reflection with an acoustic shadow, hard FR as a crescent-shaped strong reflection with an acoustic shadow, and no FR or gas without reflection. US findings of the rectum were defined as either R1 (no FR), R2 (FR), or R3 (hard FR) (Figure 1) [21]. US was performed by two physicians: one US specialist and one non-specialist. The specialist was a gastroenterologist with experience from over 100 intestinal ultrasound tests, while the non-specialist was a gastroenterologist with no experience in intestinal ultrasound. Both the specialist and the non-specialist were blinded to the results of CT and DRE. The two physicians performed US for each patient and compared the findings between patients without consulting each other. The US findings of the specialist were considered correct, and the concordance rates between the two physicians were calculated.

### 2.8. Outcome Measurements

The primary endpoint was the percentage of patients diagnosed with R2 + R3. The secondary endpoint was calculated by comparing the percentage of patients diagnosed with FR by CT and DRE vs. FR diagnosis by the HUD, the percentage of patients matching the FR diagnosis by CT and DRE, and the percentage of the defecation status of patients diagnosed with R1 vs. R2 + R3. A subanalysis was performed to examine the background factors in patients diagnosed with R2 + R3.

### 2.9. Statistical Analysis

Data are expressed as means and standard deviations. Analyses were performed using the JMP statistical program (version 15.0; SAS Institute, Cary, NC, USA). In all cases, a *p* value of <0.05 was regarded as statistically significant without adjustment for multiple testing. Missing data were analyzed without imputation. As this was an exploratory observational study, feasibility was prioritized, and cases accumulated during the study period were included. Therefore, the sample size was not predesigned. FR diagnosis and concordance rates were calculated as percentages. Patients were divided into two groups, R1 (the group that did not have FR) and R2/R3 (the group that had FR), and a univariate analysis was performed. A Student’s *t*-test was used for the univariate analysis. Thereafter, we focused on the patient factors associated with a positive univariate analysis outcome (*p* < 0.05), and a multivariate analysis was performed to determine the independent factors contributing to FR in the rectum. Concordance rates were calculated as the percentage of patients who had or did not have FR by HUD that coincided with the presence or absence of FR by CT or DRE.

## 3. Results

This section is divided by subheadings. It should provide a concise and precise description of the experimental results, their interpretation, and the experimental conclusions that can be drawn.

### 3.1. Baseline Characteristics

**Figure 2:**
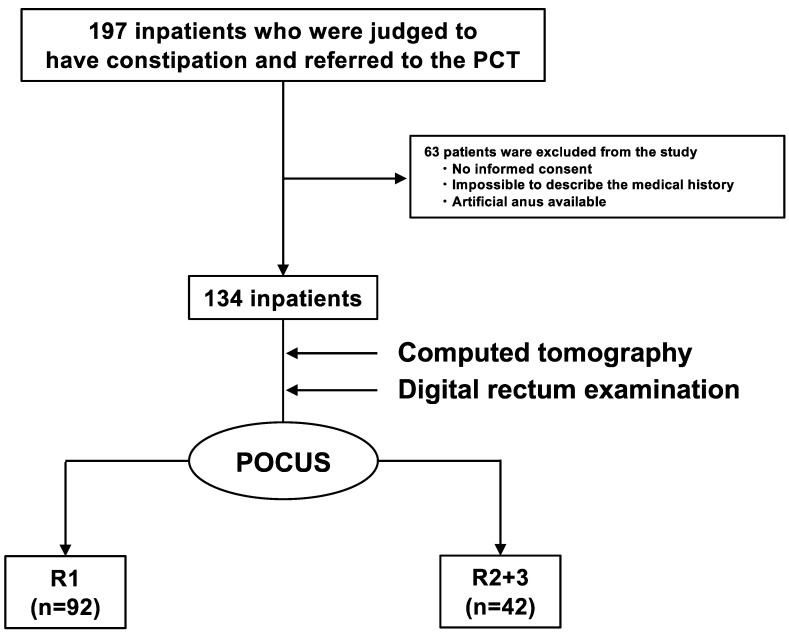
Flowchart of the observational study. Of the 197 inpatients who were judged to have constipation and were referred to the PCT, 63 were excluded, and 134 were enrolled. POCUS was performed on 134 inpatients using HUDs. They were divided into two groups (R1 (the group that did not have FR) and R2 + R3 (the group that had FR)). There were 92 R1 and 42 R2 + R3 inpatients. PCT: palliative care team, POCUS: point-of-care ultrasound, HUD: handheld ultrasound device, FR: fecal retention.

Of the 197 inpatients referred to the PCT, 63 were excluded based on the previously mentioned exclusion criteria, and 134 were enrolled (Figure 2). Patient characteristics are summarized in Table 1. The average age of the patients was 68 ± 11 years, and there were 64 men (48%). There were 128 (96%) patients with cancer and 6 (4%) patients without cancer. The primary cancer sites were the head and neck in 29 (22%) patients, the hepatobiliary region and the pancreas in 27 (20%), and the gastrointestinal tract in 18 (13%). Seventy-five patients (56%) had undergone or were undergoing treatment (51 in chemotherapy, two in radiotherapy, 18 in chemoradiotherapy, one in the preoperative period, and three in the postoperative period), and 59 patients (44%) were receiving best supportive care. In total, 27 (20%) and 35 (26%) patients had histories of constipation and surgery, respectively. Further, 75 (56%) patients were taking opioids, 63 (47%) were taking constipation drugs, and 11 (8%) were taking diuretics. A total of 62 (46%) patients had chronic constipation that met the Rome IV criteria, and 47 (34%), 74 (55%), and 13 (11%) had BSFS values of 1–2, 3–5, and 6–7, respectively. Additionally, 61 patients (46%) experienced a sensation of incomplete evacuation, and 55 patients (41%) experienced a loss of defecation desire.

### 3.2. Percentage of Constipation Patients with Fecal Retention

The rectum was visualized in all 134 patients (100%). Based on the presence or absence of FR in the 134 patients assessed using the HUD, there were 92 (69%) R1 and 42 (31%) R2 + R3 patients (Table 1). In addition, the concordance rate between the specialist and the non-specialist for R1, R2, and R3 was 93%, and the concordance rate between the two physicians for R1 and R2 + R3 diagnoses was 97% (Table 1).

### 3.3. Usefulness of the HUD as the Gold-Standard Method for Diagnosis of FR by CT and DRE

**Figure 3:**
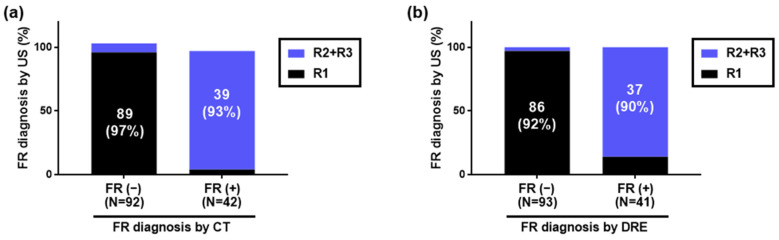
Diagnosis of fecal retention in the rectum using handheld ultrasound devices. Of 134 inpatients, 42, 42, and 41 were diagnosed with FR by HUDs, CT, and DRE, respectively, while 92, 92, and 93 were diagnosed with no FR, respectively. (**a**) Comparison with FR diagnosis by CT. Of those diagnosed with FR by CT, 39/42 (93%) were diagnosed with FR by HUDs. Of those diagnosed with no FR by CT, 89/92 (97%) were diagnosed with no FR by HUDs. (**b**) Comparison with FR diagnosis by DRE. Of those diagnosed with FR by DRE, 37/41 (90%) were diagnosed with FR by HUDs. Of those diagnosed with no FR by DRE, 86/93 (92%) were diagnosed with no FR by HUDs. FR: fecal retention, HUD: handheld ultrasound device, CT: computed tomography, DRE: digital rectal examination.

Using HUDs, CT, and DRE, 42 (31%), 42 (31%), and 41 (31%) patients were diagnosed with FR, while 92 (69%), 92 (69%), and 93 (69%) were diagnosed with no FR, respectively. A graph comparing those diagnosed with or without FR by CT to those diagnosed with or without FR by HUDs is shown in Figure 3a. Additionally, a graph comparing those diagnosed with or without FR by DRE to those diagnosed with or without FR by HUDs is shown in Figure 3b. 

**Figure 4:**
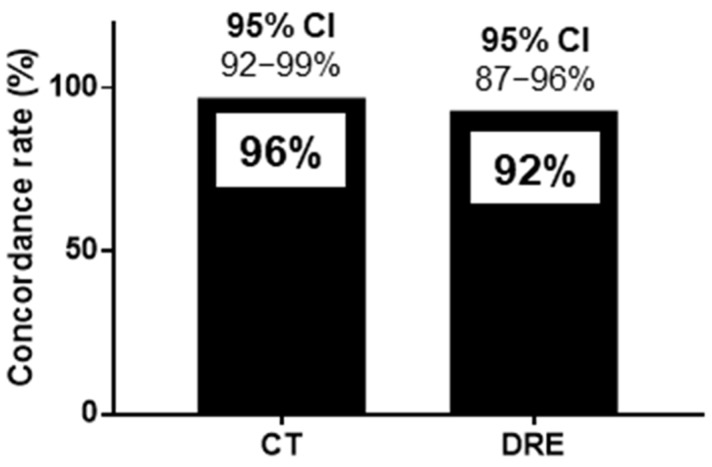
Concordance rate for handheld ultrasound devices and computed tomography and handheld ultrasound devices and digital rectal examination for the diagnosis of fecal retention in the rectum. The concordance rates for FR diagnosis were 128/134 (96%) for CT and HUDs and 123/134 (92%) for DRE and HUDs. CI: confidence interval, CT: computed tomography, DRE: digital rectal examination, FR: fecal retention, HUD: handheld ultrasound device.

The sensitivity, specificity, positive predictive value, negative predictive value, and positive likelihood ratio of HUDs for CT were 93%, 97%, 93%, 97%, and 28.5, respectively. Those of HUDs for DRE were 88%, 94%, 86%, 95%, and 13.5, respectively (Table 2). The concordance rates for FR diagnosis were 128/134 (96%) for CT and HUDs and 123/134 (92%) for DRE and HUDs, as shown in Figure 4. The 95% confidence interval (CI) for the sensitivity of HUDs for CT was 85%-100%, and that for specificity was 93%–100% (Figure 4). The 95% CI for the sensitivity of HUDs for DRE was 78%–98%, and that for specificity was 88%–99%. The 95% CI for the concordance rates for CT and HUDs was 92%–99%, and that for DRE and HUDs was 87%–96% (Figure 4).

### 3.4. Risk Factors for Patients with FR

We investigated the background risk factors for FR (Table 3). In the univariate analysis, age ≥ 65 years, Eastern Cooperative Oncology Group Performance Status 3–4, a history of constipation, surgical operations, and use of diuretics were significantly different in the R1 and R2 + R3 groups. Additionally, the multivariate analysis revealed that a history of constipation, surgical operations, and use of diuretics were significantly different between the two groups. We investigated the risk factors for the defecation status of patients with FR (Table 4). In the univariate analysis, there were significant differences in straining, BSFS values of 1–2, a sensation of incomplete evacuation, difficulty in defecation, SBM < 3 times/week, and loss of defecation desire in the R2 + R3 cases compared to the R1 cases. In the multivariate analysis, BSFS values of 1–2 and the sensation of incomplete evacuation were significantly different between the two groups.

## 4. Discussion

To the best of our knowledge, this study is the first to demonstrate that a HUD is useful for FR diagnosis in patients with constipation in palliative care settings while also extracting risk factors for patients with FR. The gold-standard techniques—CT imaging and DRE—were used to diagnose FR. While CT scanning is a highly useful and sensitive diagnostic tool, allowing clear, continuous visualization of stool characteristics inside the colon [22,23,24,25,26], it has radiation exposure issues. Furthermore, because palliative care settings usually involve patients with a poor overall performance status, transporting them to the CT imaging center is challenging. DRE also has some disadvantages owing to psychological factors unrelated to its diagnostic effectiveness [27]. In this study, the concordance rate of an FR diagnosis by HUDs was 96% for CT and 92% for DRE. This suggests the usefulness of HUDs for diagnosing FR. The slightly lower concordance rate with DRE compared to that with CT may be because DRE depends on the length of the finger, making it difficult to palpate the entire rectum.

The previous study noted that ultrasonography could classify FR through expert consensus meetings, but this had not been validated in patients in settings such as palliative care. Therefore, the main difference between this study and the previous report is that data were collected prospectively from patients in palliative care to evaluate the diagnostic performance of ultrasonography for constipation.

In palliative care settings, patients are often unaware of symptoms due to a poor health condition, and an early constipation diagnosis by the HUD allows for prompt therapeutic intervention. Regarding the risk factors associated with FR, constipated patients with hard stools (BSFS values of 1–2) and the sensation of incomplete evacuation were more likely to have FR. Therefore, HUDs may be useful diagnostic tools for patients who can be interviewed regarding the stool shape and presence or sensation of incomplete evacuation.

Constipation is a common side effect of opioid use, but this study did not find a significant difference in opioid use between the two groups. The reason for this may be that in the palliative care setting of this study, there were many R1 patients whose stools did not move to the rectum due to the suppression of gastrointestinal peristalsis. Although this study was performed in patients with constipation in a palliative care setting, we believe that opioid-induced constipation (OIC) should be diagnosed in patients with OIC who meet the Rome IV C6 criterion.

In this study, the concordance rate between the specialist and the non-specialist who performed POCUS for R1 and R2 + R3 diagnoses was 97%, indicating that it may be possible for non-specialists to diagnose FR easily. Additionally, HUD equipment is inexpensive and simple; hence, it can be used by physicians, nurses, and laboratory technicians, and educational programs for nurses have been developed [17]. Furthermore, research is being carried out on artificial-intelligence-based echographic diagnosis to help standardize the diagnosis of constipation by echography [28]. The prevalence of constipation in palliative care patients varies in different surveys according to the patient population assessed and the definition of constipation used, with overall prevalence estimates ranging from 32% to 87%, suggesting the need for standardized and specific methods [29,30,31,32].

Constipation in patients with serious illness may also be associated with nausea, early satiety, decreased oral intake, delirium, rectal ulceration, and gastrointestinal tract perforation; hence, early diagnosis of constipation is important. However, many patients in palliative care settings have a poor overall performance status and limited communication abilities, making ultrasound devices important for objectively diagnosing FR. Palliative care settings have become widespread in hospitals, palliative care units, and home-visit clinics, which comprise multidisciplinary teams including physicians, nurses, laboratory technicians, and pharmacists. In nursing, handheld echography-based constipation treatments have proven useful in the home-visit setting [33,34]. If the diagnosis of constipation is standardized, physicians, nurses, and laboratory technicians may manage constipation by providing prompt treatment to patients. Therefore, we believe that it is important to promote the use of HUDs for constipation with FR among physicians and the nursing staff. Currently, research related to HUDs is being conducted in the nursing field, and an educational system for nursing is being developed, providing promise for the future of the field.

This study had several strengths. It demonstrated the usefulness of HUDs for diagnosing FR in patients with constipation in a palliative care setting and used a simple and inexpensive portable ultrasound device. Moreover, this study used CT and DRE as the gold standards for FR diagnosis. However, this study also had some limitations. It was conducted at a single institution and did not include hospital settings, palliative care units, or home visits, excluding a population of palliative care patients. Additionally, the study population comprised a single race in Japan, and the echographic diagnosis was performed only by physicians. There was also a selection bias in the patient population with constipation for those who met the Rome IV criteria.

In conclusion, the HUD was useful for diagnosing FR in patients with constipation in a palliative care setting within the study. The findings suggest that HUDs could provide valuable support for appropriate treatment selection. Further multicenter studies with larger sample sizes are warranted. Further, future studies should investigate the percentage of patients with FR in palliative care settings and the differences in the diagnostic abilities of physicians and nurses regarding the diagnosis of FR using HUDs. It will be desirable to develop a constipation treatment algorithm based on rectal ultrasonographic findings in the future.

## Figures and Tables

**Table 1:** Ultrasonographic findings, demographic characteristics, and defecation status of inpatients referred to the palliative care team.

Variables		Patients
		(*n* = 134)
Ultrasonographic finding, *n* (%)		
	R1	92 (68)
	R2	33 (25)
	R3	9 (7)
	R2 + R3	42 (31)
	Ascites no/yes	125/9 (93/7)
Diagnosis concordance rate of R1, R2, and R3 (%)		93
Diagnosis concordance rate of R1 and R2 + R3 (%)		97
Patient demographics		
	Male/Female, *n* (%)	64/70 (48/52)
	Age, mean (SD)	68 (11)
	Age, <65/>65 years, *n* (%)	43/91 (32/68)
	Patients with cancer/without cancer, *n* (%)	128/6 (96/4)
	Primary tumor site, *n* (%)	
	Head and neck	29 (22)
	Hepatobiliary, pancreas	27 (20)
	Gastrointestinal tract	18 (13)
	Abdominal disease/other	75/59 (56/44)
	Undergone or Undergoing treatment/BSC, *n* (%)	75/59 (56/44)
ECOG-PS, *n* (%)		
	0/1/2/3/4	11/53/25/21/24 (8/40/19/16/17)
	0–2/3–4	89/45 (66/34)
History		
	Constipation, *n* (%)	27 (20)
	Duration of constipation history, mean (SD)	6.4 (14)
	Diarrhea, *n* (%)	2 (1)
	Duration of diarrhea history, mean (SD)	0.02 (0.2)
	Surgical operation, *n* (%)	35 (26)
Comorbidities, *n* (%)		
	Diabetes mellites	23 (17)
	Cerebral spinal cord disease	13 (10)
	Peritoneal dissemination	12 (9)
	Ascites	9 (7)
	Hypothyroidism	7 (5)
	Mental disorder	4 (3)
Regular use of medication, *n* (%)		
	Non-opioid analgesics	74 (55)
	Opioid analgesics	75 (56)
	OMEDD ≥60 mg	21 (28)
	Laxatives	63 (47)
	Magnesium oxide	45 (34)
	Stimulant laxative	8 (6)
	Naldemedine	34 (25)
	Antacid	48 (36)
	Diuretic	11 (8)
	Anticholinergic drug	5 (4)
	Antipsychotic	6 (4)
	Auxiliary drug	25 (19)
Prognosis prediction		
	PaP score, mean (SD)	4.7 (4.2)
	≥9/5.6-8.9/≤5.5, *n* (%)	25/20/89 (19/15/66)
	PPI, mean (SD)	2.9 (3.3)
	≥6.5/3.6–6.4/≤3.5, *n* (%)	18/28/88 (13/21/66)
Meals, *n* (%)		
	Appetite loss	60 (45)
	Amount of food mean (%)	63 (36)
	Oral intake reduction, normal/moderate/severe	72/29/33
	Tube feeding	14 (10)
Defecation status, *n* (%)		
	Chronic constipation based on Rome IV criteria	62 (46)
	Straining	53 (39)
	Bristol stool form scale values of 1–2/3–5/6–7	47/74/13 (34/55/11)
	Sensation of incomplete evacuation	61 (46)
	Difficulty in defecation	31 (23)
	Manual assistance with defecation	6 (4)
	SBM < 3 times/week	59 (44)
	Loss of defecation desire	55 (41)

BSC: best supportive care, ECOG-PS: Eastern Cooperative Oncology Group Performance Status, OMEDD: oral morphine equivalent daily dose, PaP: Palliative Prognosis, PPI: Palliative Prognostic Index, R1: no fecal retention, R2 + R3: fecal retention and hard fecal retention, SBM: spontaneous bowel movement, SD: standard deviation.

**Table 2:** Specific metrics of diagnostic accuracy.

	HUDs for CT	95% CI	HUDs for DRE	95% CI
Sensitivity	93	85–100	88	78–98
Specificity	97	93–100	94	88–99
Positive predictive value	93		86	
Negative predictive value	97		95	
Positive likelihood	28.5		13.5	

HUD: handheld ultrasound device, CT: computed tomography, CI: confidence interval, DRE: digital rectal examination.

**Table 3:** Demographic characteristics of inpatients referred to the palliative care team and the background risk factors for fecal retention.

		R1	R2 + R3	Univariate Analysis			Multivariate Analysis		
Patient Demographics, *n* (%)		*n* = 92	*n* = 42	*p* Value	Odds Ratio	95% CI	*p* Value	Odds Ratio	95% CI
Male		44 (48)	20 (48)	0.9					
Age ≥ 65 years		57 (62)	34 (81)	0.02	2.6	1.1–6.3			
Primary tumor site									
Abdominal disease		54 (59)	21 (50)	0.3					
BSC		37 (40)	22 (52)	0.2					
ECOG-PS 3-4		23 (25)	22 (52)	0.002	3.3	1.5–7.1			
History, *n* (%)									
	Constipation	5 (5)	22 (52)	<0.0001	19.1	6.5–56.7	<0.0001	24.3	7.5–95.6
	Diarrhea	3 (3)	0	0.2					
	Surgical operation	14 (15)	21 (50)	<0.0001	5.6	2.4–12.8	0.0003	7.2	2.4–23.5
Comorbidities, *n* (%)									
	Diabetes mellites	14 (15)	9 (21)	0.4					
	Cerebral spinal cord disease	9 (10)	4 (10)	0.9					
	Peritoneal dissemination	7 (7)	5 (12)	0.4					
	Ascites	6 (7)	3 (7)	0.9					
	Hypothyroidism	4 (4)	3 (7)	0.5					
	Mental disorder	2 (2)	2 (5)	0.4					
Regular use of medication, *n* (%)									
	Non-opioid analgesics	49 (53)	25 (59)	0.5					
	Opioid analgesics	49 (53)	26 (62)	0.3					
	OMEDD ≥ 60 mg	13 (27)	8 (31)	0.7					
	Laxatives	38 (41)	25 (60)	0.05					
	Magnesium oxide	26 (28)	19 (45)	0.053					
	Stimulant laxative	7 (7)	1 (2)	0.2					
	Naldemedine	17 (18)	15 (36)	0.2					
	Antacid	28 (30)	20 (48)	0.054					
	Diuretic	4 (4)	7 (17)	0.02	4.4	1.2–16.0	0.01	7.1	1.5–37.2
	Anticholinergic drug	2 (2)	3 (7)	0.2					
	Antipsychotic	5 (5)	1 (2)	0.4					
	Auxiliary drug	18 (20)	7 (17)	0.7					
Prognosis prediction									
	PaP score ≥ 9	14 (15)	11 (26)	0.1					
	PPI ≥ 6.5	28 (30)	18 (43)	0.2					
Meals, *n* (%)									
	Appetite loss	39 (42)	21 (50)	0.4					
	Oral intake reduction	40 (43)	22 (52)	0.9					
	Tube feeding	8 (9)	6 (14)	0.3					

Data are shown as the number (%) or mean (SD). BSC: best supportive care, CI: confidence interval, ECOG-PS: Eastern Cooperative Oncology Group Performance Status, OMEDD: oral morphine equivalent daily dose, PaP: Palliative Prognosis, PPI: Palliative Prognostic Index, R1: no fecal retention, R2 + R3: fecal retention and hard fecal retention, SD: standard deviation.

**Table 4:** Defecation characteristics of inpatients referred to the palliative care team and the risk factors for defecation with fecal retention.

	R1	R2 + R3	Univariate Analysis			Multivariate Analysis		
Defecation Status, *n* (%)	*n* = 92	*n* = 42	*p* Value	Odds Ratio	95% CI	*p* Value	Odds Ratio	95% CI
Straining	24 (26)	29 (69)	<0.0001	6.3	2.8–14.1			
Bristol stool form scale values of 1–2	17 (18)	30 (71)	<0.0001	11	4.7–25.8	0.02	5.2	1.2–26.4
Sensation of incomplete evacuation	29 (32)	32 (76)	<0.0001	7	3.0–16.0	0.01	3.8	1.4–11.3
Difficulty in defecation	12 (13)	19 (45)	<0.0001	5.5	2.3–13.0			
Manual assistance with defecation	2 (2)	4 (10)	0.056					
SBM < 3 times/week	27 (29)	32 (76)	<0.0001	7.7	3.3–17.8			
Loss of defecation desire	28 (30)	27 (64)	0.0002	4.1	1.9–8.9			

Data are shown as the number (%) or mean (SD). CI: confidence interval, R1: no fecal retention, R2 + R3: fecal retention and hard fecal retention, SBM: spontaneous bowel movement, SD: standard deviation.

## Data Availability

The authors declare that all data supporting the findings of this study are available within the article. Researchers can apply for data by submitting a proposal to the corresponding author.
